# Standardized Minimal Residual Disease Detection by Next-Generation Sequencing in Multiple Myeloma

**DOI:** 10.3389/fonc.2019.00449

**Published:** 2019-06-06

**Authors:** Qiumei Yao, Yinlei Bai, Alberto Orfao, Chor Sang Chim

**Affiliations:** ^1^Department of Medicine, Queen Mary Hospital, The University of Hong Kong, Pokfulam, Hong Kong; ^2^Institute for Immunology and School of Medicine, Tsinghua University, Beijing, China; ^3^Department of Medicine and Cytometry Service (Nucleus), Cancer Research Centre (IBMCC, USAL-CSIC) and CIBERONC, Institute for Biomedical Research of Salamanca (IBSAL), University of Salamanca (USAL), Salamanca, Spain

**Keywords:** minimal residual disease, multiple myeloma, next-generation sequencing, allele-specific oligonucleotide-PCR, sensitivity

## Abstract

Next-generation sequencing (NGS) has been applied to monitor minimal residual disease (MRD) in multiple myeloma (MM). Standardized DNA input and sequencing depth is essential for achieving a uniform sensitivity in NGS-based MRD study. Herein, the sensitivity of 10^−5^ was verified by a standardized experimental design based on triplicate measurements of 1 μg DNA input and 1 million sequencing reads using the LymphoTrack-MiSeq platform. MRD level was defined as the mean MRD burden of the triplicates. Two spike-in controls at concentrations of 0.001% tumor plasma cells (PC) for verifying the sensitivity of 10^−5^ and 0.01% (or 0.005%) for MRD normalization were systematically analyzed. The spike-in control of 0.001% MRD was consistently detected in all samples, confirming a sensitivity of 10^−5^. Moreover, this standardized NGS approach yielded MRD measurements concordant with serological response and comparable to allele-specific oligonucleotide (ASO) real-time quantitative (RQ)-PCR. Moreover, NGS showed an improved sensitivity and provided quantification of MRD for cases assigned “positive but not quantifiable” (PNQ) by ASO RQ-PCR, even without the use of patient-specific probes/primers. Issues regarding the specificity of myeloma-specific sequences as MRD target, optimal input for spike-in normalization, and interpretation of MRD from triplicates are discussed. Herein, the standardized LymphoTrack-MiSeq-based method is verified to carry a sensitivity of 10^−5^, hence an effective tool for MRD monitoring in MM. As only a small number of samples are tested here, further study with a larger number of patients is warranted.

## Introduction

Correlation between depth of response and survival has long been established in multiple myeloma (MM) (1). Novel agent combinations have resulted in high rates of complete response (CR) (2). Despite this, a major portion of CR patients early or later on will eventually relapse, suggesting that low but clinically relevant levels of minimal residual disease (MRD) remain in the majority of patients attaining CR (3). Therefore, highly sensitive techniques are required to detect deeper response than clinical CR, as recently highlighted by the International Myeloma Working Group (IMWG) (4).

Next-generation sequencing (NGS) of immunoglobulin (Ig) gene rearrangements has been applied to assess MRD and shown to be able to detect MRD at a level as low as 10^−6^ (5, 6). Moreover, MRD negativity using the 10^−5^ threshold predicted for a superior progression-free survival (PFS) and overall survival (OS) of MM patients achieving at least very good partial response (VGPR), and improved PFS when CR patients are separately considered (7). Recently, the IMWG introduced the definition of MRD-negativity as the absence of clonal PC by either sequencing- or flow cytometry-based techniques with a minimum sensitivity of <10^−5^ (4) using the LymphoSIGHT (Sequenta/Adaptative) (7) and next-generation flow (NGF) EuroFlow approaches (8) as the reference methods, respectively. This is due to the fact that the majority of studies on the prognostic value of NGS-based MRD in MM were derived from a commercial service by LymphoSIGHT platform (Sequenta/Adaptive Inc.) (7, 9, 10). More recently, MRD studies using this platform to evaluate the efficacy of daratumumab, a human IgGκ anti-CD38 monoclonal antibody, have been reported (11, 12). Notably, the sensitivity was claimed to be of at least 10^−5^ (7, 12), 10^−6^ (9, 11), or 10^−7^ (10), depending on the different studies. In parallel, the LymphoTrack assay has become available as a commercial kit adapted to the detection of MRD by NGS and has been evaluated in MM (8, 13). However, these studies failed in providing an experimental validation of the sensitivity of <10^−5^ for the LymphoTrack assay with variable (non-standardized) DNA inputs and depth of sequencing, which are essential for reproducible sensitivity among within and among distinct samples.

In this study we aimed to validate the sensitivity of 10^−5^ of a standardized workflow of LymphoTrack-MiSeq platform through detection of spike-in controls in follow-up MM samples and compare the NGS MRD results with those obtained by allele-specific oligonucleotide (ASO) real-time quantitative (RQ)-PCR.

## Materials and Methods

### Patients and Samples

Four Chinese MM patients included in this study received autologous stem cell transplantation (ASCT) after VTD induction (bortezomib–thalidomide–dexamethasone) (14) or PAD (bortezomib–doxorubicin–dexamethasone) induction, followed by consolidation therapy using an additional two cycles of VTD or not, and then thalidomide maintenance (thalidomide 50 mg daily) for 1 year. Diagnostic and follow-up bone marrow (BM) samples were studied. This study was approved by the Institutional Review Board of the University of Hong Kong/Hospital Authority Hong Kong West Cluster with informed consents. Patient and sample characteristics are shown in Table 1.

**Table 1:** Patient and sample characteristics.

**Patient ID**	**Isotype**	**β2-Microglobulin, mg/L**	**Albumin, g/dL**	**LDH high:1**	High-risk cytogenetics^*^	**Induction regimen**	**Consolidation therapy**	**Follow-up samples**		
								**ID**	**Time**	**Clinical response**
1	A	3.0	3.9	0	t (4;14);	VTD	Yes	FU-1	Before ASCT	VGPR
								FU-2	After ASCT	CR
								FU-3	After consolidation	CR
2	G	9.1	3.3	0	t (14;16); del 17p	VTD	Yes	FU-1	Before consolidation	VGPR
								FU-2	After consolidation	CR
3	D	13.6	4.2	0	No	PAD	No	FU-1	After ASCT	CR
4	Non-secretory	3.3	4.2	1	No	VTD	No	FU-1	After ASCT	CR

*LDH, lactate dehydrogenase; FU, follow-up; VTD, bortezomib–thalidomide–dexamethasone; PAD, bortezomib–doxorubicin–dexamethasone; ASCT, autologous stem cell transplantation; VGPR, very good partial response; CR, complete response*.

* *Presence of del(17p) and/or translocation t(4;14) and/or translocation t (14;16)*.

### MRD Measurements by NGS

Genomic DNA was extracted from BM samples using the QIAamp DNA Blood Mini Kit (Qiagen, Hilden, Germany). DNA concentration was measured by the Qubit dsDNA HS assay kit and Qubit 2.0 fluorometer (Thermo Fisher Scientific, Waltham, MA). DNA input was of 500 ng for clonality assessment of the four diagnostic samples. PCR amplification of Ig gene rearrangement fragments was performed using the LymphoTrack *IGH* (FR1, FR2, FR3) and *IGK* assays according to the instructions of the manufacturer. Primers in the LymphoTrack assays were designed with Illumina adapters. Subsequently, each amplicon was purified by AMPure XP beads (Beckman Coulter, Brea, CA), followed by quantification using the KAPA Library Quantification Kit (Roche, Basel, Switzerland). An equal amount of all purified amplicons was pooled into a library of 4 nM, denatured, diluted, loaded on to the MiSeq, and subjected to MiSeq run (v3, 2 × 300 cycles; Illumina, San Diego, CA). Sequencing data in FASTQ format were analyzed using the LymphoTrack software package (InVivoScribe Technologies, San Diego, CA). A clonotype was defined when at least five identical sequencing reads were obtained. Frequency of a clonotype was determined by the number of sequencing reads of the clonotype divided by the total number of sequencing reads. A myeloma clonotype for tracking MRD was defined as an identical sequence with a frequency of >5% as previously described (7).

**Figure 1:**
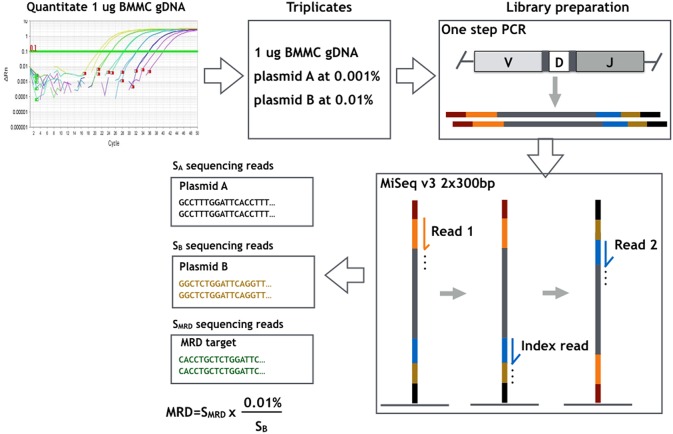
Overview of the workflow of MRD measurement method used and evaluated in the present study. The number of cells contained in 1 μg bone marrow mononuclear cells (BMMCs) of each sample was determined by real-time PCR standard curve method. Each MRD sample was studied in triplicates of 1 μg DNA input with a sequencing g depth of 1 million reads in each replicate. Two plasmids containing unique *IGH* sequences were added to each replicate: plasmid A at concentration of 10^−5^ for validation of the sensitivity of 10^−5^, and plasmid B at 10^−4^ for deriving the amplification factor, i.e., percentage of tumor alleles per sequencing read (0.01%/sequencing reads of plasmid B). MRD of each replicate was derived by multiplying patient-specific sequencing reads with the amplification factor.

For detection of MRD in the follow-up samples, triplicates of 1 μg DNA input for each sample were subjected to PCR amplification using the LymphoTrack *IGH* (FR1) assay. DNA from a healthy donor BM was used as negative control. Subsequent library preparation and sequencing were performed as described above for clonality detection in diagnostic samples. The sequencing depth for each replicate was designed to be of 1 million sequencing reads. The number of cells contained in 1 μg of each sample was validated by the real-time PCR standard curve method using plasmids, in which the albumin gene is cloned. Two plasmids containing unique *IGH* sequences were added to each replicate: one at the concentration of 10^−5^ (plasmid A: copy number equivalent to 0.001% of the number of total cells in a replicate) for validation of the sensitivity of 10^−5^, and the other at 5 × 10^−5^ or 10^−4^ (plasmid B) for obtaining an amplification factor, i.e., percentage of tumor alleles per sequence read. The MRD level in each replicate was calculated from the corresponding reads of the myeloma-specific sequence and the amplification factor. The final MRD level of a sample was defined as the mean MRD levels of the triplicates. An overview of the MRD measurement method described above is shown in Figure 1.

### MRD Study by ASO RQ-PCR

Clonality detection and subsequent MRD assessment by ASO RQ-PCR were performed as previously described (15). Clonality and the sequence of the complementarity-determining region 3 (CDR3) were identified by sequential PCR of the *IGH* VDJ, *IGH* DJ, and *IGK* VJ rearrangements, followed by Sanger sequencing. For MRD assessment, ASO forward primers with/without patient-specific reverse primers were designed. RQ-PCR was performed and interpreted according to the EuroMRD guidelines (16).

## Results and Discussion

### Assessment of Clonality in Diagnostic Samples

Myeloma-specific clonal *IGH* VDJ rearrangements present in diagnostic MM BM samples were identified by *IGH* VDJ FR1 PCR in NGS. Patient 1 had two unrelated clonal rearrangements (frequency of clonotype-1 of 45.5% and of clonotype-2 of 18.5%), while patients 2–4 had a single clonotype (frequency of 42.8, 80.3, and 13.9%, respectively). The clonotype sequences identified by NGS were exactly the same as those derived from *IGH* multiplex consensus PCR followed by Sanger sequencing, including the two clonal rearrangements present in patient 1. These two clonal rearrangements identified in patient 1 might occur in the same myeloma cell, or two independent myeloma cells, in case of biclonal disease. However, this could not be ascertained as one was a functional rearrangement, while the other was non-functional. This is consistent with the “allelic exclusion model of IgH locus rearrangements,” whereby pro-B cells with a productive V(D)J rearrangement suppress V_H_ to DJ_H_ rearrangement of the second allele (17). Moreover, this model predicts that cells that make a non-functional V_H_DJ_H_ joint on the first allele will subsequently rearrange V_H_ to DJ_H_ on the second allele, resulting in the generation of mature lymphocytes carrying non-productive V_H_DJ_H_ rearrangement on one allele, and productive rearrangement on the other (17). Results of clonality detection are shown in Table 2.

**Table 2:** Results of clonality detection.

**Patient ID**	**Clonality detected by Sanger sequencing**	**Clonality detected by NGS**				
		**V-gene**	**D-gene**	**V-gene**	**Frequency**	**CDR3 identical to Sanger sequencing**
1	Clone 1: V_H_3.9(0)-1-7-(2)D5.12(4)-2-(18)J_H_6	Clone 1 (IGHV3-9_01)	IGHD5-12_01	IGHJ6_03	45.5	100%
	Clone 2: V_H_3.13(10)-5-(7)D3.22(10)-11-(1)J_H_3	Clone 2 (IGHV3-13_01)	IGHD3-22_01	IGHJ3_02	18.5	100%
2	V_H_2.5(2)-6-(2)D1.26(2)-3-(5)J_H_4	IGHV2-5_09	IGHD1-26_01	IGHJ4_02	42.8	100%
3	V_H_3.66(0)-5-(15)D3.16(7)-7-(14)J_H_6	IGHV3-66_02	IGHD3-16_02	IGHJ6_02	80.3	100%
4	V_H_3.21(1)-9-(8)D2.21(3)-3-(5)J_H_6	IGHV3-21_02	IGHD2-21_01	IGHJ6_03	13.9	100%

*NGS, next-gene sequencing; CDR3, complementarity-determining region*.

### NGS-Based MRD Detection

#### Specificity of Sequences of Spike-In Control and MRD Target

First of all, none of the *IGH* sequences of the two plasmids added to each patient replicate sample were detected in the negative control, which used DNA from a healthy donor BM, indicating specificity of the sequences; hence, this indicates that, in principle, they were good candidates for spike-in controls. Subsequently, the feasibility of the clonal *IGH* VDJ rearrangements identified in the diagnostic samples as MRD targets in follow-up samples from the same patients was also evaluated. Clonal myeloma-specific sequences were not detected in the normal control except clonotype-1 of patient 1. The mean frequency of MRD from the triplicates of clonotype-1 identified in patient 1 were 1.5 × 10^−2^ in sample follow-up-1, 7.7 × 10^−4^ in follow-up-2, 4.3 × 10^−4^ in follow-up-3, but only 2.2 × 10^−6^ in the normal control, which is 2–4 logs lower than the level of MRD detected in the MRD follow-up samples. While false-positive MRD amplification may arise from non-specific primer binding to similar V-(D)-J sequences in normal lymphocytes, NGS MRD positivity is based on the detection of the identical myeloma clonal sequence (18). Therefore, MRD positivity in normal background in NGS might arise from normal cells producing the same sequence with myeloma cells (19). Indeed, identical IgH rearrangement with identical CDR3 sequences produced by unrelated cell populations has been observed in mice (20). However, there are 24 deleted/inserted nucleotides in the CDR3 region; hence, an identical clonotypic sequence generated by normal unrelated cells is unlikely (19). Another possibility is index misassignment when sequencing reads from pooled libraries are identified and sorted computationally by de-multiplexing before data analysis (21, 22). On the other hand, DNA contamination is less likely. As patient 1 harbored two clonotypes including clonotype 2, one would expect to find sequence of clonotype 2, in addition to clonotype 1, in the same normal control in case contamination had occurred. Moreover, while we are not able to analyze the number of tumor reads in the normal control due to the absence of a spike-in normalization plasmid, the number of reads would have ranged from 245 to 610 in sample FU-1 of patient 1 should there be one tumor cell in the FU sample. Given the small number of reads in the normal control, herein, *N* = 5, 2, and 0 in the respective replicate, we believe that index misassignment is the most likely reason.

#### Sensitivity of NGS MRD Detection

Regarding sensitivity, it should be noted that the *IGH* sequence of 10^−5^ control plasmid was detected in at least one of the triplicates of all follow-up BM samples; thus, the sensitivity of 10^−5^ was achieved in all seven samples tested. Obtained sequencing reads for 10^−5^ control plasmid in each replicate are shown in Table 3. This is the first report in which the sensitivity of 10^−5^ was verified for the LymphoTrack-Miseq platform. Although the ability to detect MRD at the level of 10^−6^ disease burden has been demonstrated by serial dilutional experiments using the LymphoSIGHT platform (5, 6), a few points remain to be clarified in these experiments. First, MRD assessment was not the same for every follow-up sample, since the number of total input cells for MRD evaluation varied substantially (e.g., between 86,143 and 1,556,654 cells) (5). Moreover, the ability to analyze a total of 10^7^ cells, which will entail analysis of between 5 and 50 μg of DNA in one PCR reaction is technically challenging, if not currently impossible. Therefore, the exact technical details need to be provided to help clarify how the experiment was conducted. Finally, as the sensitivity of an MRD assay is dependent on the number of total input cells, a sensitivity of 10^−6^ might not be guaranteed in every sample.

**Table 3:** Results of minimal residual disease measured by next-generation sequencing and allele-specific oligonucleotide real-time quantitative-PCR.

**Patient/ sample**	**Clinical response**	**Replicate**	**Input cell number**	**Total reads (million)**	**10^**−5**^ control reads**	**5** **×** **10**^****−5****^**/10**^****−4****^ **control reads (frequency)**		**MRD reads**	**MRD burden**	**Mean MRD**	**Median MRD**	**RQ-PCR MRD**
1/FU-1	VGPR	−1	3.2 × 10^5^	1.1	656	5 × 10^−5^	3,050 (2.8 × 10^−3^)	Clonotype 1: 18545 Clonotype 2: 2599	Clonotype 1: 0.030% Clonotype 2: 0.004%	Clonotype 1: 0.046% Clonotype 2: 0.011%	Clonotype 1: 0.043% Clonotype 2: 0.013%	Clonotype 1: 23 copies/10^5^ cells Clonotype 2: 45 copies/10^5^ cells
		−2	3.2 × 10^5^	1.1	387		1,226 (1.1 × 10^−3^)	Clonotype 1: 15911 Clonotype 2: 3400	Clonotype 1: 0.065% Clonotype 2: 0.014%			
		−3	3.2 × 10^5^	0.9	24		1,411 (1.5 × 10^−3^)	Clonotype 1: 12154 Clonotype 2: 3679	Clonotype 1: 0.043% Clonotype 2: 0.013%			
1/ FU-2	CR	−1	2.5 × 10^5^	0.8	0	10^−4^	13,128 (1.5 × 10^−2^)	Clonotype 1: 1737 Clonotype 2: 0	Clonotype 1: 0.0013% Clonotype 2: 0	Clonotype 1: 0.0004% Clonotype 2: 0.0006%	Clonotype 1: 0 Clonotype 2: 0	Clonotype 1: Positive, <10^−4^ Clonotype 2: Negative
		−2	2.5 × 10^5^	0.9	1,279		8,216 (0.9 × 10^−2^)	Clonotype 1: 0 Clonotype 2: 1497	Clonotype 1: 0 Clonotype 2: 0.0018%			
		−3	2.5 × 10^5^	0.5	857		9,433 (2.0 × 10^−2^)	Clonotype 1: 0 Clonotype 2: 0	Clonotype 1: 0 Clonotype 2: 0			
1/ FU-3	CR	−1	2.8 × 10^5^	2.0	11	10^−4^	702 (3.5 × 10^−4^)	Clonotype 1: 1021 Clonotype 2: 30	Clonotype 1: 0.016% Clonotype 2: 0.0004%	Clonotype 1: 0.010% Clonotype 2: 0.003%	Clonotype 1: 0.013% Clonotype 2: 0.0004%	Clonotype 1: positive, < 10^−4^ Clonotype 2: positive, < 5 × 10^−4^
		−2	2.8 × 10^5^	1.2	250		616 (5.2 × 10^−4^)	Clonotype 1: 807Clonotype 2: 10	Clonotype 1: 0.013% Clonotype 2: 0.0002%			
		−3	2.8 × 10^5^	1.7	0		407 (2.4 × 10^−4^)	Clonotype 1: 141 Clonotype 2: 333	Clonotype 1: 0.003% Clonotype 2: 0.008%			
2/FU-1	VGPR	−1	1.9 × 10^5^	1.2	0	10^−4^	65 (5.5 × 10^−5^)	0	Negative	Negative	Negative	Negative
		−2	1.9 × 10^5^	1.0	11		70 (5.5 × 10^−5^)	0	Negative			
		−3	1.9 × 10^5^	0.9	1		43 (4.8 × 10^−5^)	0	Negative			
2/FU-2	CR	−1	2.0 × 10^5^	1.1	0	5 × 10^−5^	369 (3.4 × 10^−4^)	0	Negative	Negative	Negative	Negative
		−2	2.0 × 10^5^	0.5	5		0	0	Negative			
		−3	2.0 × 10^5^	0.8	48		516 (6.7 × 10^−4^)	0	Negative			
3/FU-1	CR	−1	1.7 × 10^5^	1.4	185	10^−4^	208 (1.4 × 10^−4^)	3,547	0.170%	0.082%	0.049%	Positive, < 5 × 10^−4^
		−2	1.7 × 10^5^	2.0	8		730 (3.7 × 10^−4^)	1,962	0.027%			
		−3	1.7 × 10^5^	1.1	5		340 (3.2 × 10^−4^)	1,674	0.049%			
4/FU-1	CR	−1	1.7 × 10^5^	2.9	99	10^−4^	111 (3.8 × 10^−5^)	687	0.061%	0.042%	N/A	30 copies/10^5^ cells
		−2	1.7 × 10^5^	1.3	9		120 (9.3 × 10^−5^)	280	0.023%			
		−3	1.7 × 10^5^	2.1	7		5 (2.3 × 10^−6^)	311	N/A			
Healthy	N/A	−1	3.8 × 10^5^	0.6	N/A							
donor		−2	3.8 × 10^5^	0.9								
		−3	3.8 × 10^5^	1.6								

*FU, follow-up; VGPR, very good partial response; CR, complete response; N/A, not applicable*.

To analyze the prognostic impact or the efficacy of novel agents by MRD monitoring, some studies used pre-defined MRD cutoff levels (7, 10–12, 23), while others used MRD-detectable/undetectable as end-points (13, 24). In the latter reports, a uniform sensitivity for the MRD detection assay is important as MRD-negativity might refer to different levels of MRD in case sensitivity varies among distinct samples. Herein, we advocate each follow-up BM sample to be analyzed in triplicate with a standardized DNA input of 1 μg per replicate for MRD detection to achieve a uniform sensitivity of 10^−5^, the minimal requirement to qualify for sequencing-based MRD-negativity according to the IMWG criteria (4). In turn, the Euro-MRD guidelines for ASO RQ-PCR recommend 500 ng in triplicates for MRD assessment, whereby a sensitivity of 10^−4^ to 10^−5^ can be achieved. However, patient-specific primers/probes are often required with the ASO RQ-PCR approach, which is labor-intensive and time-consuming (15, 25).

#### Molecular Number of Spike-In Control for Normalization

Of note, with the approach here used, the MRD level was normalized by the amplification factor (percentage of tumor alleles per sequence read) obtained from the 5 × 10^−5^ or 10^−4^ diluted plasmid. The LymphoSIGHT platform applied a pool of plasmids containing three unique *IGH* clonotypes to obtain a final amplification factor (number of molecules per sequence read) for absolute quantitation of tumor alleles (5). However, copy numbers of the plasmids have not been reported. A recent MRD study using the LymphoTrack-Miseq platform used spike-in DNA corresponding to 1,000 clonal cells (13). Given the limited data in the field, the optimal copy number of spike-in positive control DNA for absolute quantitation of tumor alleles still remains to be defined. Calculation of MRD levels using spike-in controls in NGS-based MRD assays is based on the same principles of using the standard curve in ASO RQ-PCR. According to the Euro-MRD guidelines for ASO RQ-PCR, dilution points included in the standard curve for quantitation of MRD in the follow-up samples should be within the “quantitative range,” which requires the delta Ct of the three replicates to differ by < 1.5, hence equivalent to a 2.8-fold difference in copy number. At present, there is still no consensus for the spike-in control (the amplification factor) in NGS-based MRD assessment; however, it might be inferred that the concentration of this spike-in control should not be too low. In our study, concentrations of 5 × 10^−5^ and 10^−4^, corresponding to approximately 10 and 20 copies of plasmids, were assessed. Sequencing reads (frequency) of 5 × 10^−5^/10^−4^ plasmids are shown in Table 3. Indeed, our data showed that a wide variation in the frequency of plasmid reads was observed at the lower concentration of 5 × 10^−5^ among the triplicates in one of two samples. Whether a high copy number (hundreds) of spike-in control may result in an inaccurate quantitation of very low MRD levels of tumor alleles needs to be confirmed. Nevertheless, a spike-in plasmid at approximately 10^−4^ appears appropriate for MRD normalization based on our limited experimental data, as variation lower than 2.8-fold in frequency among triplicates of the 10^−4^ plasmid were achieved in four of five samples. However, in the follow-up sample of patient 4, minor variation was observed in two of three replicates. Thus, MRD in this sample was determined by those two replicates and obtained comparable MRD level with ASO RQ PCR (42 copies vs. 30 copies, per 10^5^ cells).

#### Interpretation of MRD From Triplicates

**Figure 2:**
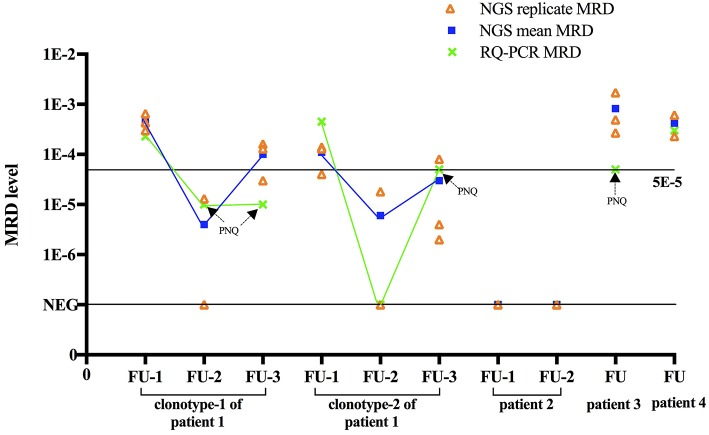
Comparison between NGS and RQ-PCR. For each MRD sample, NGS based MRD of triplicates, mean MRD and ASO RQ-PCR based MRD are shown. For comparison purposes, cases that were defined as “positive not quantifiable” in ASO RQ-PCR were placed to sensitivity of that patient (10^−5^ for clonotype-1 of patient 1, 5 × 10^−5^ for clonotype-2 of patient 1 and patients 3). Aberration: FU, follow-up; PNQ, positive not quantifiable.

Based on the approach here used, final MRD levels for a sample were derived from the mean MRD values of triplicates for that specific sample. In Euro-MRD guidelines for ASO RQ-PCR, an MRD result for a given follow-up sample might be interpreted as either quantifiable, positive but not quantifiable (PNQ), or negative (16). In NGS-based MRD assessment, MRD positivity is defined as the presence of two identical reads of the MRD target in the LymphoSIGHT platform and five identical reads in the LymphoTrack-Miseq approach. The percentage of MRD reads in a positive sample is calculated with the absolute myeloma molecules normalized by the spike-in control divided by total input cells in the reaction. Previously reported NGS MRD studies (7, 9–13, 23, 26) did not refer usage of MRD replicate measurements in follow-up samples; thus, the concept of PNQ for interpretation of MRD results has not yet been established in NGS-based MRD assays. Meanwhile, quantitative discrimination in NGS was regarded as super-imposable to its sensitivity, in contrast to ASO RQ-PCR, in which MRD levels beyond the quantifiable range are assigned “PNQ” (26). Indeed, a certain MRD level can be reached in MRD samples as long as tumor-specific alleles are detected by NGS. Moreover, an improved quantitative ability (greater sensitivity) is observed in NGS compared to RQ-PCR. For instance, Faham et al. reported that LymphoSIGHT was highly quantitative for frequencies above 10^−5^ (5), while frequencies of 10^−4^ to 10^−5^ were usually detectable but not quantifiable in ASO RQ-PCR. Here, a higher quantifiable range of NGS compared to ASO RQ-PCR was observed for the first follow-up sample of patient 1. For MRD assessment by ASO RQ-PCR, ASO forward primers and patient-specific reverse primers were designed for clonotypes 1 and 2, achieving a quantitative range of 10^−4^ and 5 × 10^−4^, respectively. If similar criteria of quantitative range in ASO RQ-PCR (i.e., delta Ct of the three replicates to differ by no more than 1.5) are applied to NGS (i.e., 2.8-fold difference in frequency in triplicates), the spike-in control of 5 × 10^−5^ in this same could be regarded as quantifiable. However, MRD measured from a single replicate by NGS is not necessarily accurate. In fact, random errors increased at clonotype frequencies below 10^−5^ in the serial dilutional experiments performed by Faham et al. (5). Similarly, triplicates applied in our study also clearly showed that despite residual disease was detected at very low MRD levels of 10^−5^, they were not reproducible among replicates, consistent with Poisson statistics for low numbers of target molecules, a limitation that would potentially be overcome by increasing the DNA input. Therefore, we applied triplicates of 1 μg DNA for MRD measurement for each follow-up sample and MRD level was defined as mean MRD burden of the triplicates. Results of measured MRD are shown in Table 3 and Figure 2.

### Comparison Between NGS and ASO-PCR to Evaluate MRD

In the four patients here studied by NGS, MRD was also analyzed in parallel by ASO RQ-PCR using patient-specific primers, rendering a sensitivity of 5 × 10^−5^ to 10^−5^. As a result, MRD was detected by ASO RQ-PCR in the five follow-up samples of patients 1, 3, and 4 but not in the two follow-up samples of patient 2. Notably, NGS yielded MRD+ or MRD– results concordant with ASO RQ-PCR in all these seven follow-up samples.

The dynamic change of MRD in the three sequential MRD-positive samples obtained by NGS and ASO-PCR was fully concordant. The serological response of patient 1 was VGPR for the follow-up-1 sample and CR for the follow-up-2 and follow-up-3 samples. Correspondingly, MRD of clonotype 1 was reduced from 0.045% in sample follow-up-1 to PNQ (positive but non-quantifiable as MRD level is below the quantitative range, 10^−4^, of this patient) in samples at follow-up-2 and−3 by ASO RQ-PCR, and reduced from 0.046 to 0.0004% and 0.010% by NGS (Figure 2). Therefore, both methods showed the expected decrease of MRD levels associated with the change in serological response. In addition, clonotype 2 of patient 1 was detected in the follow-up-2 sample by NGS but not by ASO RQ-PCR. This would be attributed to the higher sensitivity of NGS (10^−5^) compared to ASO RQ-PCR (5 × 10^−5^) for clonotype 2. Ladetto et al. compared MRD in sequential follow-up samples in MM by NGS and ASO RQ-PCR, and discordances were observed in 44% (20/45) of the samples (26). Among the 20 discordant cases, ASO RQ-PCR yielded a positive result in 8 that were negative by NGS MRD, indicating that this discordance could not be accounted for by the different sensitivity between the two approaches. Therefore, further studies are needed to understand the reasons for the discordant MRD results obtained with NGS and ASO RQ-PCR, as discordant MRD results post-treatment by these two techniques would generate different conclusions about the MRD response.

In summary, this report provides the first data on a standardized protocol for MRD assessment using the LymphoTrack-Miseq platform based on the use of triplicates of 1 μg DNA input for each MRD sample and a sequencing depth of 1 million sequencing reads per sample. In addition, in the proposed protocol, two spike-in controls were also included: one serving as a 10^−5^ sensitivity marker and the other as a reference for MRD normalization. Moreover, the standardized NGS protocol proposed yielded MRD results comparable to ASO RQ-PCR, both of which were concordant with the serological response. Furthermore, NGS proved to achieve an improved sensitivity and more reproducible quantification of low levels of MRD, otherwise declared PNQ by ASO RQ-PCR, without the need for patient-specific probe/primers and, hence, a less labor-intensive technique and faster turn-around time. As only a small number of samples are tested here, further study with a larger number of patients is warranted.

## Ethics Statement

This study was carried out in accordance with the recommendations of the Institutional Review Board of the University of Hong Kong/Hospital Authority Hong Kong West Cluster with informed consents. All subjects gave written informed consent in accordance with the Declaration of Helsinki. The protocol was approved by the Institutional Review Board of the University of Hong Kong/Hospital Authority Hong Kong West Cluster.

## Author Contributions

CC designed and supervised the study. QY and YB performed the experiments. All authors analyzed the data, wrote the manuscript, and approved the final version.

### Conflict of Interest Statement

The authors declare that the research was conducted in the absence of any commercial or financial relationships that could be construed as a potential conflict of interest.

## Abbreviations

NGS: next-generation sequencing
Ig: immunoglobulin
MRD: minimal residual disease
MM: multiple myeloma
PC: plasma cell
ASO: allele-specific oligonucleotide
RQ: real-time quantitative
PNQ: positive but not quantifiable
CR: complete response
PFS: progression-free survival
OS: overall survival
VGPR: very good partial response
NGF: next-generation flow
ASCT: autologous stem cell transplantation
BM: bone marrow
CDR3: complementarity-determining region.

## References

[1] Kapoor P Kumar SK Dispenzieri A Lacy MQ Buadi F Dingli D. Importance of achieving stringent complete response after autologous stem-cell transplantation in multiple myeloma. J Clin Oncol. (2013) 31:4529–35. 10.1200/jco.2013.49.0086 24248686 PMC3871514

[2] Mailankody S Korde N Lesokhin AM Lendvai N Hassoun H Stetler-Stevenson M. Minimal residual disease in multiple myeloma: bringing the bench to the bedside. Nat Rev Clin Oncol. (2015) 12:286–95. 10.1038/nrclinonc.2014.239 25622976 PMC7712493

[3] Paiva B van Dongen JJ Orfao A. New criteria for response assessment: role of minimal residual disease in multiple myeloma. Blood. (2015) 125:3059–68. 10.1182/blood-2014-11-568907 25838346 PMC4513329

[4] Kumar S Paiva B Anderson KC Durie B Landgren O Moreau P. International myeloma Working Group consensus criteria for response and minimal residual disease assessment in multiple myeloma. Lancet Oncol. (2016) 17:e328–e46. 10.1016/s1470-2045(16)30206-6 27511158

[5] Faham M Zheng J Moorhead M Carlton VE Stow P Coustan-Smith E. Deep-sequencing approach for minimal residual disease detection in acute lymphoblastic leukemia. Blood. (2012) 120:5173–80. 10.1182/blood-2012-07-444042 23074282 PMC3537310

[6] Logan AC Zhang B Narasimhan B Carlton V Zheng J Moorhead M. Minimal residual disease quantification using consensus primers and high-throughput IGH sequencing predicts post-transplant relapse in chronic lymphocytic leukemia. Leukemia. (2013) 27:1659–65. 10.1038/leu.2013.52 23419792 PMC3740398

[7] Martinez-Lopez J Lahuerta JJ Pepin F Gonzalez M Barrio S Ayala R. Prognostic value of deep sequencing method for minimal residual disease detection in multiple myeloma. Blood. (2014) 123:3073–9. 10.1182/blood-2014-01-550020 24646471 PMC4023416

[8] Flores-Montero J Sanoja-Flores L Paiva B Puig N Garcia-Sanchez O Bottcher S. Next generation flow for highly sensitive and standardized detection of minimal residual disease in multiple myeloma. Leukemia. (2017) 31:2094–103. 10.1038/leu.2017.29 28104919 PMC5629369

[9] Avet-Loiseau H Lauwers-Cances V Corre J Moreau P Attal M Munshi N Minimal residual disease in multiple myeloma: final analysis of the IFM2009 trial. Blood. (2017) 130 (Suppl 1):435.

[10] Takamatsu H Takezako N Zheng J Moorhead M Carlton VEH Kong KA. Prognostic value of sequencing-based minimal residual disease detection in patients with multiple myeloma who underwent autologous stem-cell transplantation. Ann Oncol. (2017) 28:2503–10. 10.1093/annonc/mdx340 28945825 PMC5834061

[11] Dimopoulos MA White DJ Benboubker L Cook G Leiba M Morton J Daratumumab, lenalidomide, and dexamethasone (DRd) versus lenalidomide and dexamethasone (Rd) in relapsed or refractory multiple myeloma (RRMM): updated efficacy and safety analysis of pollux. Blood. (2017) 130(Suppl 1):739.

[12] Mateos MV Dimopoulos MA Cavo M Suzuki K Jakubowiak AJ Knop S Phase 3 randomized study of daratumumab plus bortezomib, melphalan, and prednisone (D-VMP) versus bortezomib, melphalan, and prednisone (VMP) in newly diagnosed multiple myeloma (NDMM) patients (Pts) ineligible for transplant (ALCYONE). Blood. (2017) 130(Suppl 1):LBA−4-LBA.

[13] Medina A Jiménez C Puig N Sanchez-Vega B Flores-Montero J González M New alternatives for the evaluation of minimal residual disease (MRD) detection by next generation sequencing in multiple myeloma. Blood. (2017) 130 (Suppl 1):1783.

[14] Chim CS Lie AK Chan EY Liu HS Lau CW Yip SF Treatment outcome and prognostic factor analysis in transplant-eligible Chinese myeloma patients receiving bortezomib-based induction regimens including the staged approach, PAD or VTD. J Hematol Oncol. (2012) 5:28 10.1186/1756-8722-5-28 22682027 PMC3418573

[15] Bai Y Wong KY Fung TK Chim CS. High applicability of ASO-RQPCR for detection of minimal residual disease in multiple myeloma by entirely patient-specific primers/probes. J Hematol Oncol. (2016) 9:107. 10.1186/s13045-016-0336-4 27724958 PMC5057274

[16] van der Velden VH Cazzaniga G Schrauder A Hancock J Bader P Panzer-Grumayer ER Analysis of minimal residual disease by Ig/TCR gene rearrangements: guidelines for interpretation of real-time quantitative PCR data. Leukemia. (2007) 21:604–11. 10.1038/sj.leu.2404586 17287850

[17] Jung D Giallourakis C Mostoslavsky R Alt FW. Mechanism and control of V(D)J recombination at the immunoglobulin heavy chain locus. Ann Rev Immunol. (2006) 24:541–70. 10.1146/annurev.immunol.23.021704.115830 16551259

[18] Kotrova M van der Velden VHJ van Dongen JJM Formankova R Sedlacek P Bruggemann M. Next-generation sequencing indicates false-positive MRD results and better predicts prognosis after SCT in patients with childhood ALL. Bone Marrow Transplant. (2017) 52:962–8. 10.1038/bmt.2017.16 28244980

[19] Moppett J Wakeman S Duez M Bartram J Wright G Hubank M Immunoglobulin/T-cell receptor (Ig/TCR) allele usage in normal and on treatment bone marrow samples in childhood acute lymphoblastic leukaemia—implications for NGS based MRD analysis. Blood. (2016) 128:4073.

[20] Seidl KJ MacKenzie JD Wang D Kantor AB Kabat EA Herzenberg LA. Frequent occurrence of identical heavy and light chain Ig rearrangements. Int Immunol. (1997) 9:689–702. 9184914 10.1093/intimm/9.5.689

[21] Li Q Zhao X Zhang W Wang L Wang J Xu D. Reliable multiplex sequencing with rare index mis-assignment on DNB-based NGS platform. BMC Genom. (2019) 20:215. 10.1186/s12864-019-5569-5. 30866797 PMC6416933

[22] Effects of Index Misassignment on Multiplexing and Downstream Analysis (white paper). Available online at: https://www.illumina.com/content/dam/illumina-marketing/documents/products/whitepapers/index-hopping-white-paper-770-2017-004.pdf

[23] Martinez-Lopez J Sanchez-Vega B Barrio S Cuenca I Ruiz-Heredia Y Alonso R. Analytical and clinical validation of a novel in-house deep-sequencing method for minimal residual disease monitoring in a phase II trial for multiple myeloma. Leukemia. (2017) 31:1446–9. 10.1038/leu.2017.58 28210002 PMC5467041

[24] Cheng S Inghirami G Cheng S Tam W. Simple deep sequencing-based post-remission MRD surveillance predicts clinical relapse in B-ALL. J Hematol Oncol. (2018) 11:105. 10.1186/s13045-018-0652-y 30134947 PMC6103872

[25] Bai Y Orfao A Chim CS. Molecular detection of minimal residual disease in multiple myeloma. Br J Haematol. (2018) 181:11–26. 10.1111/bjh.15075 29265356

[26] Ladetto M Bruggemann M Monitillo L Ferrero S Pepin F Drandi D. Next-generation sequencing and real-time quantitative PCR for minimal residual disease detection in B-cell disorders. Leukemia. (2014) 28:1299–307. 10.1038/leu.2013.375 24342950

